# The Symptom Flatulence and Its Symptomatic Treatment

**Published:** 1915-07

**Authors:** George M. Niles

**Affiliations:** Gastroenterologist to the Georgia Baptist Hospital, Wesley Memorial Hospital, Atlanta Hospital; Consulting Gastroenterologist to the Anti-Tuberculosis Association, etc., Atlanta, Ga. 922 Candler Building.


### THE SYMPTOM FLATULENCE AND ITS SYMPTOMATIC TREATMENT.

By George M. Niles, M. D.

Gastroenterologist to the Georgia Baptist Hospital, Wesley Memorial Hospital, Atlanta Hospital; Consulting Gastroenterologist to the Anti-Tuberculosis Association, etc., Atlanta, Ga.

Few are the disturbances of digestion that do not include among their subjective or objective manifestations either gastric or intestinal flatulence.

While this symptom is more pronounced and annoying in the nervous, it is at times a source of material discomfort to the most phlegmatic. The writer, therefore, feels that any suggestions tending to a better understanding of some of the underlying causes, as well as the relief of the flatulence itself, will not be amiss.

Many of the patients who seek assistance for supposed heart disease, are in reality suffering from the primary or secondary effects of gastric flatulence. In some individuals the heart tolerates considerable upward pressure from a distended stomach. In the neurotic, however, this distension, or any appreciable collection of flatus in the intestines, either disturbs the cardiac action or greatly upsets the patient's mental equilibrium.

The pressure of gas in the stomach is a bane to many. It cannot be accounted for by fermentation of food products, for it is frequently produced in the presence of a sufficiency of hydrochloric acid and where no fermentation can exist. Besides, no fermentation could produce the immense quantities which are so explosively eructated by these nervous people.

One lady informed the writer that she counted the number of eructations after a light meal of toast and tea, and that they numbered one hundred and forty-one.

When uncomfortable gastric flatulence occurs in neurotic people, when the eructations are frequent, explosive, and without much taste or odor, the trouble may generally be ascribed to aerophagia or unconsciously swallowed atmospheric air.

Aerophagia should not be confounded with the gaseous eructations accompanying extreme dilatation of the stomach, for in this condition they are of a decided odor, and contain sulphuretted hydrogen and marsh gas. It should also be distinguished from the burning eructations of hyperchlorhydria, as well as real fermentation sometimes occurring in achylic stomachs.

Vanderhoof records a case of aerophagia in a hysterical subject, where she belched over five thousand times in twenty-four hours, and the amount of air eructated being measured, was found to exceed 200 litres. It is evident that this quantity of air is many times in excess of that which could be produced by any conceivable process of fermentation; and furthermore, this air has been collected and analyzed by several investigators who have shown that it approaches in composition atmospheric air, the small content of carbon dioxid being derived from the decomposition of carbonates in the food or the alkaline saliva.

Carmimatives will generally be needed in the management of these cases, but the main point is to convince the patient that if composure is exercised, and quietude is sought during and after meals, less air will be swallowed, and most of that present will be unconsciously absorbed and expired with the breath.

Some years ago there consulted the writer a middle-aged maiden lady of high intelligence who complained bitterly of a flatulent condition of her stomach, which would generally come on after supper, especially if the day had been a busy or trying one. When she felt this gas, she would walk the floor and indulge in various gymnastic exercises, believing that she must get rid of it before she could safely retire. Upon an explanation of the real condition, with the assurance that if she would sit quietly and placidly, the gas would either pass into the intestine or be absorbed, she made an honest effort and soon found to her surprise that there was no gas to expel. In her case she was swallowing the air as fast as or faster than she could eject it, in the meanwhile provoking her nervous system almost into a frenzy.

Probably this condition is always associated with a certain lack of tone in the gastric musculature, and in individuals with neurotic stigmata it is presumable that certain states of nervousness produce an inhibition of the nerves controlling this normal tone. Certain emotions or reflex agencies bring about a loss of tone in the walls of the stomach exactly analagous to the inhibition of the vasoconstrictor nerves which produce blushing. In this way, in nervous conditions; there is produced a potential relaxation of the stomach walls, so that the organ is easily inflated by air swallowed with the food or between meals.

At the same time that the explosive eructations are going on, some of the air may be forced through the pylorus, giving rise to intestinal flatulence.

A most annoying trouble due to a neurosis is the passage of air through the intestines, and the accompanying rumbling denominated borborygmus. This is increased under emotional stress like earophagia, while the anxiety over it increases it still more. Old men seldom complain of this to their physicians, but middle-aged or elderly women find it a source of keen embarrassment. The older female sufferers are often stout, with relaxed and incompetent abdominal walls, so that the empty intestines do not fall together as they should, but rather tend to lie apart, allowing spaces between which the atonic abdominal walls "balloon," thus favoring an accumulation of gas. Often after these patients have been exercising to the point of fatigue, and sit down in a warm room, the expansion of air in the intestines quickly leads to rumbling there. This experience is quite common with elderly people in cold weather, and the odor of the flatus, when passed, is but slightly offensive.

In young women, when troubled with flatulence or borborygmus it often makes them so nervous and leads to such dread, that it hinders their participation in social usages, makes them fear to associate with any but their immediate family, and occasions the most poignant mental distress. Some young women suffer from rumbling in the intestines whenever more than four hours have passed since their last meal. This phenomenon is more likely to manifest itself when they are with people whom they desire to impress favorably. Dread and fear play a large part in this nervous rumbling, and it is probably due to an exaggeration of peristalsis, and the crowding into large intestinal spaces small collections of air that under normal peristalsis would escape from one portion of the gut to another without audible sound.

Moral suasion and encouragement, with the injunction always to eat a light lunch when the slightest sensation of emptiness is felt, will greatly ameliorate this embarrassing condition. It is often necessary to give in addition an alkaline carminative, and to enforce sane hygienic regulations, as there is frequently, a material aspect that psychotherapy alone will not control.

Many individuals, especially women, who adopt a sedentary occupation and lose in weight, suffer from both borborygmus and sensitiveness of the intestines. The same management as that just suggested will generally prove satisfactory.

The flatulence from gall-stones, a diseased gall-bladder, or a diseased appendix; that accompanying gastric or duodenal ulcer, or cancer of the stomach or intestines demands obvious means for relief. The moderate flatulence following abdominal operations—all these call for a rational line of therapy. All these forms, however, can be mitigated, pending operative or material measures of treatment, and it is due the patient that efforts be put forth to relieve the annoyances of flatulence.

In addition to various well-recognized procedures, as gentle cathartics, enemas, hot local applications, hydrotherapy, etc. the following carminative prescriptions are recommended:

℞

Spts. anisi . . . . . . . . . . . . . . . . . . . . . . . . . . . . . . . . . . dr. ii
Resorcinolis . . . . . . . . . . . . . . . . . . . . . . . . . . . . . . . . gr. xv
Lactis magnesiae q. s. ad. . . . . . . . . . . . . . . . . . . . . . . . . oz. iv

Sig. One or two teaspoonfuls as needed for flatulence.

℞

Spts. foeniculi . . . . . . . . . . . . . . . . . . . . . . . . . . . . . . . dr. ii
Resorcinolis . . . . . . . . . . . . . . . . . . . . . . . . . . . . . . . . gr. xv
Lactis magnesiae q. s. ad. . . . . . . . . . . . . . . . . . . . . . . . . oz. iv

Sig. One or two teaspoonfuls as needed for flatulence.

Where, in stagnation, there is great fermentation and much excess of organic acids, this may be used:

℞

Creosoti (beechwood) . . . . . . . . . . . . . . . . . . . . . . . . gr. viii
Sodii bicarb . . . . . . . . . . . . . . . . . . . . . . . . . . . . . . . . dr. ii
Pv. acaciaee . . . . . . . . . . . . . . . . . . . . . . . . . . . . . . . . dr. i
Spts. lavandulae co. . . . . . . . . . . . . . . . . . . . . . . . . . . dr. i
Aquae q. s. ad. . . . . . . . . . . . . . . . . . . . . . . . . . . . . . . oz. iii

Sig. One teaspoonful after meals.

In the presence of nausea combined with flatulence and heartburn, of which some patients complain so bitterly, employ this:

R

Spts. amygdalae amare ........................dr. ii
Resorcinolis ..................................gr. xx
Lactis magnesiae q. s. ad.........................oz. iv

Sig. One teaspoonful after meals, and repeat as needed. When there is painful flatulence, this is suggested:

R

Orthoformi ...................................a a dr. ii
Bismuthi subcarbonatis.
Mist. cretae co. g. s. ad. .........................oz. iv

As a stimulating carminative, a small amount of spirits of ginger may be included, or where a decided carminative is needed, the milk of asafetida, either alone, or in equal parts with the milk of magnesia, may be given at frequent intervals.

The foregoing suggestions are based, not on theory, but on experience, and are confidently offered by the writer.

922 Candler Building.

